# Compound Heterozygous *FKTN* Variants in a Patient with Dilated Cardiomyopathy Led to an Aberrant α-Dystroglycan Pattern

**DOI:** 10.3390/ijms23126685

**Published:** 2022-06-15

**Authors:** Anna Gaertner, Lidia Burr, Baerbel Klauke, Andreas Brodehl, Kai Thorsten Laser, Karin Klingel, Jens Tiesmeier, Uwe Schulz, Edzard zu Knyphausen, Jan Gummert, Hendrik Milting

**Affiliations:** 1 Erich und Hanna Klessmann-Institut für Kardiovaskuläre Forschung und Entwicklung, Klinik für Thorax- und Kardiovaskularchirurgie, Herz und Diabeteszentrum NRW, Universitätsklinikum der Ruhr-Universität Bochum, Georgstr. 11, 32545 Bad Oeynhausen, Germany; lidiapaul88@gmail.com (L.B.); bklauke@hdz-nrw.de (B.K.); abrodehl@hdz-nrw.de (A.B.); jens.tiesmeier@muehlenkreiskliniken.de (J.T.); uwe.schulz@helios-gesundheit.de (U.S.); jgummert@hdz-nrw.de (J.G.); 2 Zentrum für Angeborene Herzfehler, Herz und Diabeteszentrum NRW, Universitätsklinikum der Ruhr-Universität Bochum, Georgstr. 11, 32545 Bad Oeynhausen, Germany; tlaser@hdz-nrw.de (K.T.L.); knypoeyn@posteo.de (E.z.K.); 3 Kardiopathologie, Institut für Pathologie und Neuropathologie, Universitätsklinikum Tübingen, Liebermeisterstraße 8, 72076 Tübingen, Germany; karin.klingel@med.uni-tuebingen.de

**Keywords:** cardiogenetics, cardiomyopathy, dystroglycanopathy, fukutin, heart failure

## Abstract

Fukutin encoded by *FKTN* is a ribitol 5-phosphate transferase involved in glycosylation of α-dystroglycan. It is known that mutations in *FKTN* affect the glycosylation of α-dystroglycan, leading to a dystroglycanopathy. Dystroglycanopathies are a group of syndromes with a broad clinical spectrum including dilated cardiomyopathy and muscular dystrophy. In this study, we reported the case of a patient with muscular dystrophy, early onset dilated cardiomyopathy, and elevated creatine kinase levels who was a carrier of the compound heterozygous variants p.Ser299Arg and p.Asn442Ser in *FKTN*. Our work showed that compound heterozygous mutations in *FKTN* lead to a loss of fully glycosylated α-dystroglycan and result in cardiomyopathy and end-stage heart failure at a young age.

## 1. Introduction

Dystroglycanopathies are a group of syndromes with a broad clinical spectrum [1,2]. α-dystroglycan is a highly glycosylated protein that binds to the extracellular part of β-dystroglycan as well as to components of the extracellular matrix, such as laminin, agrin, and perlecan [3]. The protein–protein interaction with laminin is compromised by defects in glycosylation of α-dystroglycan [2]. As a complex series of steps is needed for the mature glycosylation pattern of α-dystroglycan [4,5], mutations within more than 17 genes may lead to dystroglycanopathies [1,6]. The gene *FKTN* encodes fukutin, a 461-amino acid protein with a predicted molecular weight of 53.7 kDa that has features typical of many glycosyltransferases [7,8]. Fukutin belongs to a family of enzymes involved in the modification of cell surface molecules such as glycoproteins and glycolipids [9]. No glycosyltransferase activity has been reported for fukutin. However, mutations in *FKTN* were shown to affect the glycosylation of α-dystroglycan, leading to a dystroglycanopathy [2,10]. Fukutin is a type II membrane protein localized in the Golgi apparatus [11]. It is a ribitol 5-phosphate transferase that uses cytidine diphosphate (CDP)-ribitol to generate together with the fukutin-related protein a ribitol 5-phospate tandem structure that is essential for the functions of α-dystroglycan [12]. As fukutin is expressed in many tissues including heart, brain, skeletal, muscle, and pancreas, the disease phenotypes caused by *FKTN*-mutations vary from dilated cardiomyopathy (DCM) and/or muscular dystrophy (MD)/dystroglycanopathy to severe congenital MD with brain malformation, intellectual disability, and abnormal eye structure [7,13,14]. According to the Online Mendelian Inheritance in Man database (https://www.ncbi.nlm.nih.gov/omim, accessed on 3 May 2022), the phenotypes cardiomyopathy, dilated, 1X (Phenotype MIM-number (PMIM) 611615), and the muscular dystrophy-dystroglycanopathies type A4 (congenital with brain and eye anomalies), type B4 (congenital without mental retardation), and type C4 (limb-girdle) (MDDGA4 (PMIM 253800), MDDGB4 (PMIM 613152), MDDGC4 (PMIM 611588)) are associated with *FKTN* mutations. Genetic variants in *FKTN* lead to a recessive inheritance of the disease since both alleles have to be affected for the onset of the clinical phenotype. The genetic etiology might be clinically misinterpreted as a non-familial and thus as an acquired disease, since both parents will be clinically unaffected. The severity of disease seems to be dependent on the type of mutation (nonsense, frame shift, or missense) [13,14,15]. In most cases, a nonsense mutation is combined with a missense mutation, commonly leading to a severe clinical phenotype. In cases of homozygous or compound heterozygous missense mutations, a milder phenotype with limb-girdle muscular dystrophy (LGMD) and/or DCM without intellectual disability is observed [16,17]. A common feature of all *FKTN*-mutation carriers is elevated creatine kinase (CK) plasma levels [18,19].

In this study, we reported the case of a male patient diagnosed with muscular dystrophy, DCM (heart transplanted at the age of 19 years), and elevated CK levels, who is the carrier of two *FKTN* missense variants. One missense variant was identified in each of the healthy parents, suggesting a recessive inheritance. The same combination of *FKTN* variants (NM_006731) c.895A>C (p.Ser299Arg) and c.1325A>G (p.Asn442Ser) were identified in a German patient before [20]. The patient was diagnosed with LGMD, mild dysphagia, and respiratory distress, but no cardiac involvement was reported in this patient [20]. In our case, we demonstrated a predominant cardiac involvement in the compound heterozygous mutation carrier and showed an aberrant α-dystroglycan glycosylation in the explanted myocardium of the patient.

## 2. Results

### 2.1. Compound Heterozygous FKTN-Genotype Presumably Led to Cardiomyopathy in a Young Patient

**Figure 1:**
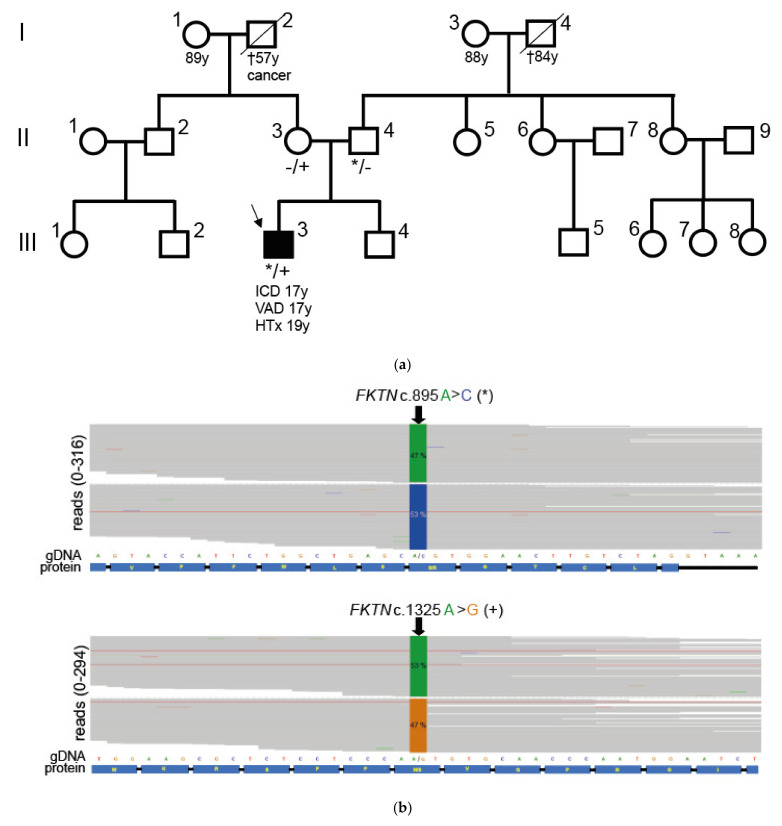

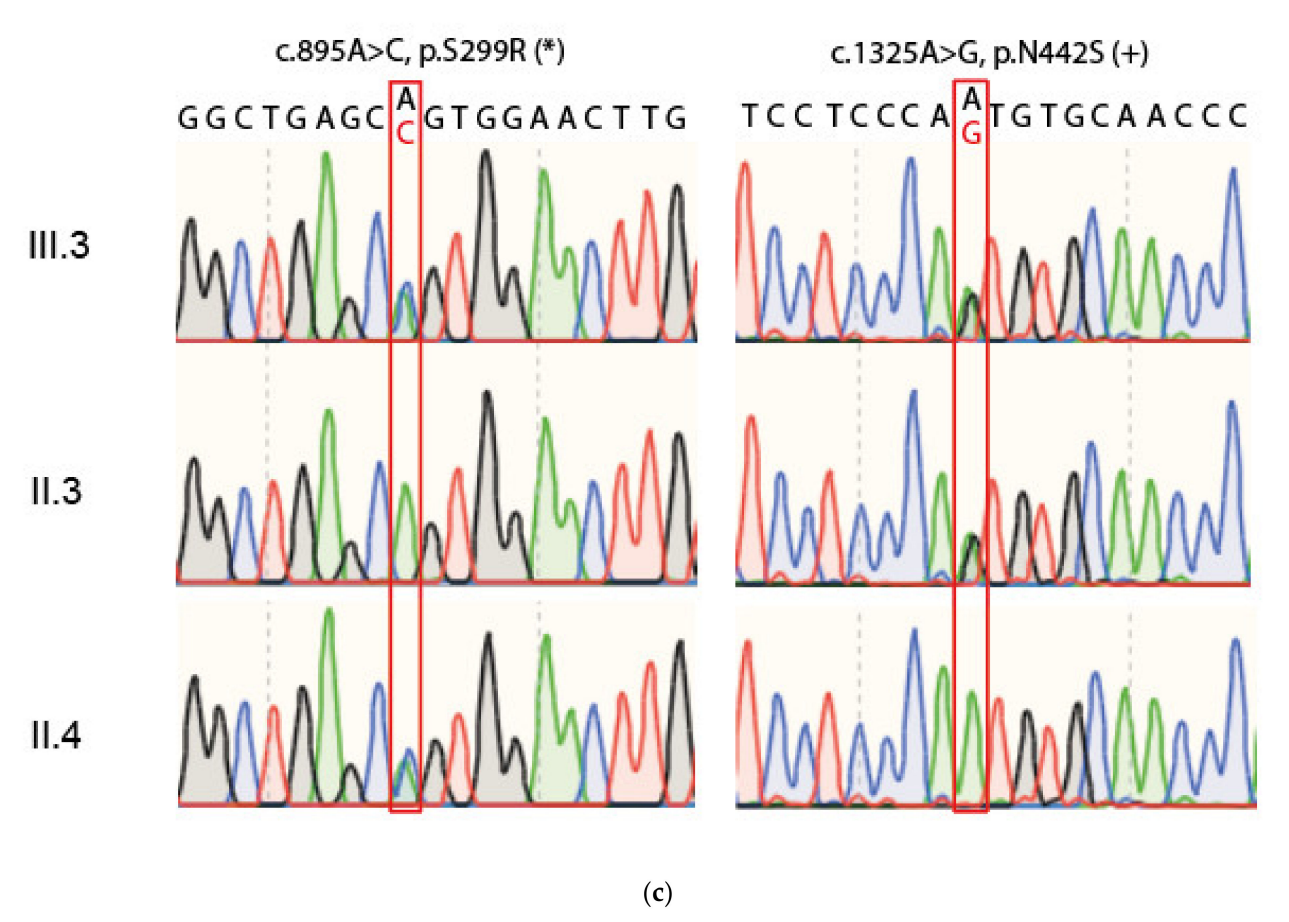
Pedigree and genotyping analysis. (**a**) Shown is the pedigree of the patient with the compound heterozygous *FKTN* variants. Circles represent females, and squares represent males. Affected individuals are shown as black-filled symbols. The index patient is marked with an arrow. † = deceased. The age of the patient at the time of ventricular assist device implantation (VAD), implantable cardioverter defibrillator implantation (ICD), or heart transplantation (HTx) is indicated (y = years). (**b**) Integrated genome view of parts of *FKTN* showing the variants identified in the index patient (III.3). The percentage of reads corresponding to the base identified at the relevant positions (c.895 and c.1325) is given (green = A, blue = C, orange = G). Below the reads, the genomic DNA sequence (gDNA) and the sequence of the translated protein (protein) are shown. (**c**) Electropherograms obtained by Sanger sequencing of the *FKTN*-regions with the particular variants. The index (III.3) patient carries both *FKTN* c.895A>C (p.S299R, *) and the c.1325A>G (p.N442S, +) variants (*/+ in A). The patient’s parents (II.3 and II.4) were heterozygous for one of the mutations (−/+ or */− in A).

We identified the compound heterozygous *FKTN*-genotypes c.895A>C p.Ser299Arg and c.1325A>G p.Asn442Ser in a German patient with muscular dystrophy, cardiomyopathy, and elevated CK levels (III.3, Figure 1).

All variants identified in the patient with a minor allele frequency (MAF < 0.0005) according to GnomAD [20] are shown in Table S1. This *FKTN*-genotype was recently identified in another German patient with muscular dystrophy [21]. In the GnomAD database [20], the variant p.Ser299Arg (rs367662190) has an allele frequency of 0.000007. The variant p.Asn442Ser (rs1429464723) has an allele frequency of 0.00002 in GnomAD [20] (version 3.1.2, 17 March 2022). The parents of the index patient (II.3 and II.4 in Figure 1) were carriers of each of the missense variants and clinically unaffected. Due to the genotype of the parents, it could be concluded that the index patient is a compound heterozygous carrier of *FKTN* p.Ser299Arg and p.Asn442Ser and that the two mutations are not localized on the same allele. The patient’s brother had no signs of cardiomyopathy and was not available for genotyping. There was no history of cardiomyopathy in the patient’s family (Figure 1a).

### 2.2. Neuromuscular Disease and Elevated CK Values in a Young Patient with Dilated Cardiomyopathy

**Figure 2:**
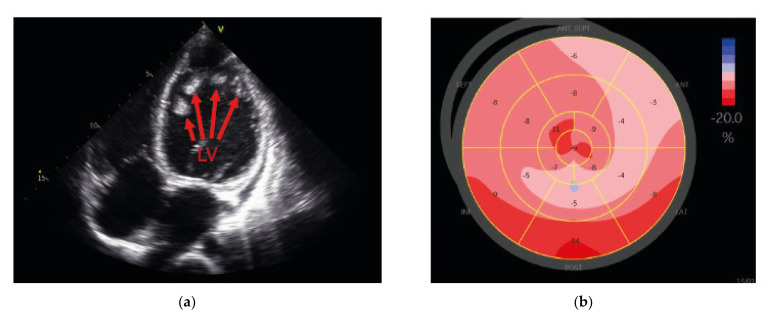

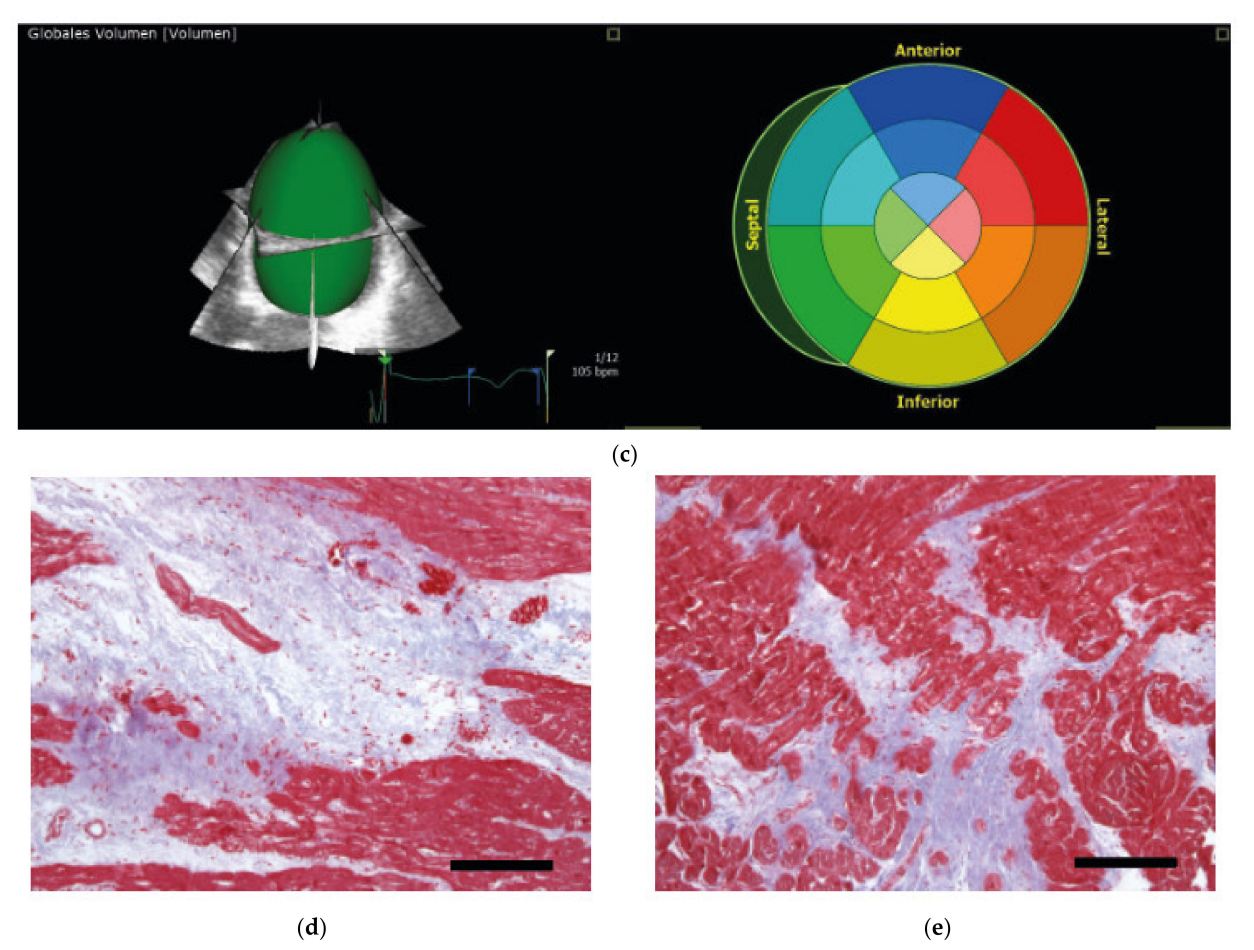
Echocardiographic data of the index patient under clinical deterioration before VAD therapy (**a**–**c**) and histochemical analysis of the explanted heart (**d**,**e**). (**a**) Four-chamber view showing the dilated left ventricle (LV) with intracavitary thrombotic material (red arrows). (**b**) Deformation analysis of global longitudinal strain values using 2D speckle tracking imaging with markedly impaired left ventricular average strain of–7.8%. (**c**) 3D-echocardiography of a LV full-volume dataset using Tomtec LV-Analysis 3 for volumetric assessment. The LV is enlarged more than twice the normal size (end-diastolic volume = 294.6 mL and end-diastolic volume index = 181.0 mL/m^2^) with a reduced ejection fraction of 20.7%. (**d**,**e**) Masson’s trichrome staining of myocardial tissue obtained from the explanted heart. Pronounced interstitial fibrosis is observed in the left (**d**) and the right (**e**) ventricles. Scale bars = 200 µm.

Anamnestically, the patient (III.3 in Figure 1a) was diagnosed with neuromuscular disease and elevated CK values at 6 years old. Using an MRI scan, the patient was diagnosed with a slightly increased left ventricular (LV) diameter (52 mm) and a reduced LV ejection fraction (52%) at 13 years old. At the age of 15 years, the analysis of a skeletal muscle biopsy revealed muscle-fiber size variability, atrophic muscle fibers, necrosis, and fibrolipomatosis, which are consistent with myodystrophy. Western blot and immunofluorescence analyses revealed reduced α-dystroglycan, dystrophin, and sarcoglycan expressions (data not shown). At 17 years old, a left ventricular assist device (VAD) and a dual chamber implantable cardioverter-defibrillator (ICD) were implanted. Echocardiographic examination before VAD-implantation showed intracavitary thrombotic material in the LV (Figure 2a), markedly impaired LV average strain (Figure 2b) and an enlarged LV with a reduced ejection fraction of 20.7% (Figure 2c). At 18 years old, the patient had an embolic middle cerebral artery infarction inter alia with global aphasia, apraxia, neglect, and right-sided facial nerve paralysis. He was heart transplanted at 19 years old. After heart transplantation (HTx), the CK values were still increased up to 3270 U/µL (norm values 0–171 U/µL). Seven years after HTx, the CK values were still >1000 U/µL. At 26 years old, the patient showed thoracolumbar scoliosis and muscular hypothrophy of the legs and drop foot. He had no other physical limitations. A histopathologic analysis of the heart at the time of explantation showed a focal and partially perivascular fibrosis (Figure 2d,e (LV) + (RV)). Of note, the majority of cardiomyocytes revealed a regular structure, and only a few cardiomyocytes were degenerated. A mild lymphocyte- and macrophage-associated inflammation was present in the LV and RV. The molecular pathological examination revealed the presence of parvovirus B19 (PVB19) DNA in the myocardium of the index patient (3000 viral copies/µg isolated nucleic acid at the time point of VAD implantation and 230 viral copies at the time of heart transplantation), likely explaining the mild immune cell infiltration.

### 2.3. FKTN Mutations Led to Aberrant α-Dystroglycan Pattern in Human Explanted Myocardium

**Figure 3:**
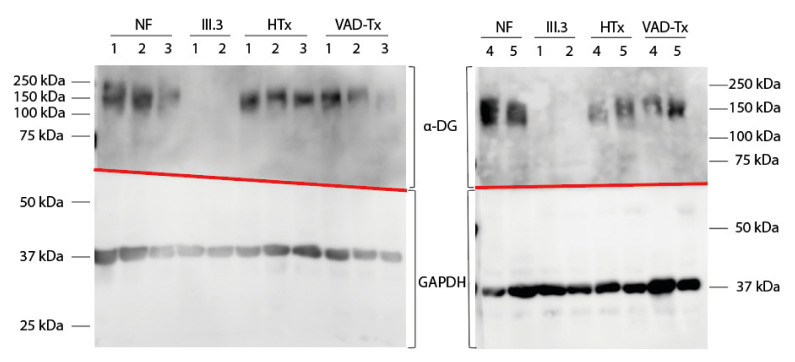
Western blot detection of α-dystroglycan (α-DG) and glyceraldehyde-3-phosphate dehydrogenase (GAPDH) in left ventricular tissue. Tissue samples from the index patient at the time of VAD implantation (III.3, 1) or at the time of VAD explantation (III.3, 2) were used. Five donor hearts (NF), five samples obtained from cardiomyopathy patients at heart transplantation (HTx), and five samples received after VAD explantation from cardiomyopathy patients (VAD-Tx) were used as controls. According to the literature, α-DG was expected to be 140 kDa and GAPDH was expected to be 37 kDa. GAPDH was observed as expected in all samples. An appropriate signal for α-DG was observed in all controls (NF, HTx, and VAD-Tx) but not in the myocardial tissue of the index patient (III.3). Fuzzy bands in the α-DG part of the Western blot were caused by differential glycosylation of α-dystroglycan.

**Figure 4:**
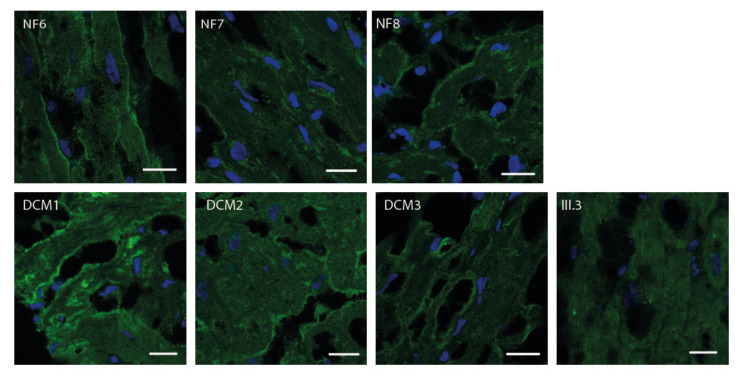
Immunofluorescence analysis of α-DG in the left ventricle. Left ventricular cryosections were labelled using an anti-α-DG primary antibody (clone VIA4-1) and an Alexa Fluor^®^ 488 conjugated goat anti-mouse secondary antibody (green). Nuclei were stained with DAPI (blue). Scale bars = 20 µm. DCM = samples from patients with dilated cardiomyopathy, NF = samples from rejected donor hearts, and III.3 = index patient (compare Figure 1). Samples from NF and DCM hearts showed a pronounced fluorescence signal around the cells. In III.3, only a weak and diffuse fluorescence signal was observed.

As it is known that *FKTN* mutations lead to aberrant α-dystroglycan glycosylation, we analyzed the α-dystroglycan pattern in the patient’s explanted LV tissue by Western blot and immunofluorescence analyses and compared it with control myocardium (Figure 3 and Figure 4).

Western blot analysis (Figure 3) revealed absence of the α-dystroglycan signal in the patient’s tissue. The lack of signal was not due to reduced total protein content, since the signal for GAPDH was comparable in all samples. The apparent molecular mass of α-dystroglycan in the heart was expected to be 140 kDa [22]. Our Western blot analysis showed an α-dystroglycan signal between 133 and 172 kDa, with a mean at 158 kDa in the age-matched controls (NF, HTx, and VAD-Tx).

Immunfluorescence analysis of the human myocardium revealed a pronounced α-dystroglycan-labelling around the cells, presumably corresponding to the basal lamina (Figure 4). The α-dystroglycan distribution in the LV sections was comparable between samples obtained from patients with DCM and rejected donor hearts (NF). In the LV tissue of the patient carrying the mutations in *FKTN* (III.3), only weak and diffuse signals were observed.

### 2.4. Molecular Modelling Showed Aberrant Interactions for the p.Ser299Arg-FKTN-Mutant

**Figure 5:**
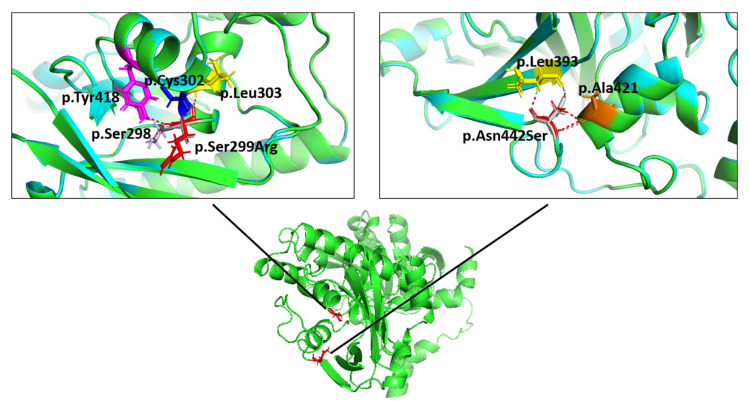
Molecular modelling of the *FKTN* variants p.Ser299Arg (left panel) and p.Asn442Ser (right panel), with structures calculated using ColabFold [23]. The influence of the variants on the molecular structure was analyzed using PyMOL Molecular Graphics System (version 2.5.2, Schrodinger LLC, New York, NY, USA). The protein backbone is shown in green for the wildtype and in cyan for the respective mutant. No major structure change was observed in the alignment between the wildtype and mutant proteins, as evidenced by the protein backbone overlay. The polar contacts were calculated using PyMOL and are shown as grey or red dashed lines for the wildtype or mutant proteins, respectively. The serine at position 299 (left panel, p.Ser299, grey) has polar contacts to p.Ser298 (purple), p.Cys302 (blue), and p.Leu303 (yellow). In the p.Ser299Arg mutant (left panel, red), an additional polar contact to Tyr418 (magenta) was observed. The asparagine and serine at position 442 (right panel, grey: p.Asn442, red: p.Ser442) had polar contacts to p.Ala421 (orange) and p.Leu393 (yellow).

Structures for wildtype and mutant fukutin were calculated with ColabFold [23]. Structures with the highest per-residue confidence metric (predicted Local Distance Difference Test, pLDDT with 100 = most confident) were chosen (wildtype: pLDDT = 92.19, p.Ser299Arg: pLDDT = 92.17, and p.Asn442Ser: pLDDT = 91.69) and aligned pairwise (Figure 5). In the p.Ser299Arg mutant, an additional polar contact for residue 299 was observed, compared with the wildtype. In the p.Asn442Ser mutant, no changes of the polar contacts for residue 442 were observed between the mutant and wildtype. No major structure changes were detected in the alignment between the wildtype and mutant proteins.

## 3. Discussion

The dystrophin-glycoprotein complex is a structure that is responsible for the linkage of the intracellular cytoskeleton to the extracellular basement membrane [24]. Within this, complex intracellular dystrophin is linked by dystroglycan to the extracellular matrix [3]. α-dystroglycan is a highly glycosylated protein, and its specific O-mannosyl glycan serves as the binding site for laminin and other extracellular matrix proteins [25,26,27]. A glycosylation deficiency can dissolve the Dystrophin Glycoprotein Complex (DGC)-extracellular matrix connection, resulting in a MD [22]. Many of the affected patients might develop cardiomyopathies with dilated ventricles and myocardial dysfunction [28]. Mutations in *FKTN*-encoding fukutin, a Golgi-based ribitol phosphate transferase [12], might cause α-dystroglycanopathy in combination with cardiomyopathy [19].

In our study, we showed that compound heterozygous mutations in *FKTN* led to cardiomyopathy, resulting in early end-stage heart failure with persisting elevated CK levels, even after HTx. Although the same combination of *FKTN* variants was identified in a 32-year-old female patient with LGMD before [21], no cardiac involvement was reported in this patient. α-dystroglycanopathies have a wide spectrum of clinical symptoms [29]. In this study, we showed that even the same combination of mutations in *FKTN* might be associated with varying phenotypes as the patient described in our study, in contrast with the patient described by Smogavec et al. [21] developed an early onset cardiomyopathy and, in spite of presenting with elevated CK levels, showed no physical limitations due to myopathy. It is known that fukutin acts as a ribitol phosphate transferase, necessary for adding the first ribitol phosphate in a tandem of ribitol phosphates that serve as the basis for the subsequent glucuronic acid-xylose repeats which bind to laminin and are recognized by the IIH6 antibody [30]. As Western blot analysis using the IIH6 antibody generated no signal in the tissue of the patient with the compound heterozygous *FKTN* variants in comparison to the control samples, it can be concluded that, in the patient’s tissue, no α-dystroglycan with glucuronic acid-xylose repeats were detected, and consequently no functional fukutin was available to produce the basis for these repeats. This strengthens the assumption that the ribitol-phosphate modification is indispensable for the functional maturation of α-dystroglycan.

It has been shown that hypoglycosylation of α-dystroglycan is revealed by loss of VIA4-1 antibody immunoreactivity, and mutations in congenital muscular dystrophies-causing glycosyltransferases lead to the hypoglycosylation and reduced laminin-binding activity [2,31,32,33]. We also observed an altered VIA4-1 signal in the myocardial cryosections of the index patient, in contrast with control samples. The signal in the patient’s sample was reduced and showed only a diffuse pattern, whereas in the control samples, the signal was localized in structures presumably corresponding to the cell membrane. This means that, in the index patient, the α-dystroglycan was either not localized at the membrane or that it was hypoglycosylated, since the used antibody does not recognize hypoglycosylated α-dystroglycan.

Application of three-dimensional molecular modelling with calculated fukutin structures [23] revealed a change in polar contacts for the p.Ser299Arg mutant (Figure 5). However, since the major structure of the mutants showed a high degree of alignment with the wildtype protein, it still remains unclear which effects the observed change will have on the enzyme activity or localization of fukutin.

Using high throughput sequencing, many novel gene variants can be identified. Nevertheless, most of these variants have to be classified as variants of unknown significance, as long as experimental evidence regarding their functional impact or cosegregation analysis is lacking in the majority of cases. Particularly in the case of recessive mutations, when a family history of disease is missing, it is difficult to predict if a homozygous or compound heterozygous variant combination is causative/pathogenic. The application of functional studies allows a direct estimation of the functional impact of a variant. Although both *FKTN* variants identified in the index patient are known from the literature, they could be classified only as variants of unknown significance using ACMG criteria (p.Ser299Arg: PM2, PP3, p.Asn442Ser: PM2, PP3) [34] since no functional data were currently available. Since our Western blot (Figure 3) and immunofluorescence analyses (Figure 4) revealed a damaging effect on α-dystroglycan, we used this as an additional ACMG criterion (PS3) and reclassified the variants p.Ser299Arg and p.Asn442Ser in combination as likely pathogenic. The correct identification of (likely) pathogenic variants is essential for genetic counselling of the patients and for the development of potential therapeutic approaches in the future.

Currently, no specific treatments for dystroglycanopathies are available. Nevertheless, since 2010, treatment strategies based on the molecular pathological mechanism are proposed [35]. These include adeno-associated viral vector gene replacement [36,37,38], gene-editing approaches using Clustered Regularly Interspaced Short Palindromic Repeats (CRISPR)-Cas9 gene editing combined with homology-directed repair [39], application of steroids [40,41], or administration of antisense nucleotides [42]. Interestingly, ribitol supplementation therapy has been shown to result in restoration of α-dysroglycan glycosylation and shows therapeutic effects in muscular dystrophy in *Fkrp*-mutant mice [43]. In 2021, a clinical trial (NCT04800874) has started testing ribitol (BBP-418) for muscular dystrophy, limb-girdle, type 2I (LGMD2I) patients (https://www.ncbi.nlm.nih.gov/omim, accessed on 10 June 2022). As *FKRP* encodes fukutin-related protein, which is the second ribitol-phosphate transferase involved in the modification of α-dystroglycan besides fukutin, a ribitol supplementation might also be discussed in patients with *FKTN* mutations if residual enzyme activity is present in the mutant fukutin. All potential therapeutic approaches show that the development of a treatment strategy for dystroglycaopythies first needs the identification of the causative gene, highlighting the relevance of our study.

Consequently, our work showed that compound heterozygous mutations in *FKTN* lead to a loss of fully glycosylated α-dystroglycan and result in cardiomyopathy and end-stage heart failure at a young age.

## 4. Materials and Methods

### 4.1. Clinical Examination of the Patients

The patient underwent comprehensive clinical examinations at the Heart and Diabetes Centre NRW (Bad Oeynhausen, Germany), including 12-lead electrocardiogram, echocardiography, and magnetic resonance imaging (MRI) examination.

### 4.2. Genetics

Molecular genetics was performed after oral and written informed consent. The local ethics committee approved the study protocol (Reg.-No. 21/2013). Genomic DNA was isolated from white blood cells using standard techniques (High Pure PCR Template Preparation Kit^®^, Roche Diagnostics GmbH, Mannheim, Germany). Panel sequencing (174 genes) was applied for variant screening in the index patient using the TruSight™ Cardio gene panel (Illumina, San Diego, CA, USA) as previously described [44]. For variant annotation, the software VariantStudio™ version 3.0 (Illumina, San Diego, CA, USA) was used. Only variants with a MAF (GnomAD [20]) <0.0005 that passed the filtering criteria were considered. Evidence criteria for mutation classification and sequencing were applied as previously published [45,46]. Variants of interest were verified by Sanger sequencing (BigDye^®^ Terminator version 1.1 Cycle Sequencing Kit, ABI PRISM^®^ 3100 genetic analyzer, Applied Biosystems, Foster City, CA, USA). Family members were checked for the *FKTN* variants found in the index patient by Sanger sequencing. The variants were classified according to the ACMG guidelines [34]. The MAFs of the variants (PM2), published data [21], and bioinformatic prediction tools (PP3) were considered for variant classification.

### 4.3. Myocardial Tissue

Myocardial tissue samples from the left ventricle were obtained from the proband’s explanted heart or during VAD implantation. After removal from the patient, the samples were immediately snap-frozen in liquid nitrogen and stored at −80 °C. As controls, samples from eight cardiomyopathy patients obtained at HTx (HTx1-5, DCM1-3), five post-VAD-implantation samples (VAD-Tx1-5), and eight non-failing donor hearts rejected for technical reasons (NF1-8) were used. The local ethics committee approved the study protocol (Reg.-No. 13/2009 and 21/2013). In addition, corresponding heart tissue probes were fixed in 4% formaldehyde and investigated by routine histology and immunohistology for visualizing immune cells [47]. For the detection of interstitial fibrosis, Masson’s trichrome staining was used.

### 4.4. Protein Extraction and Western Blot

Proteins were extracted from the human myocardial tissue using RIPA buffer (150 mM NaCl, 1 mM EDTA, 50 mM Tris-HCl, 1% (*v*/*v*) Nonidet P40 Substitute (Merck, Darmstadt, Germany), 0.25% (*w*/*v*) sodium deoxycholate, 1 mM NaF, 1 mM Na3VO4, proteinase inhibitor cocktail P2714 (Sigma-Aldrich, St. Louis, MO, USA), pH 7.4) as described previously [48]. We mixed 20–50 mg myocardial tissue with RIPA buffer (10 µL buffer/1 mg tissue) and homogenized for 60 s with the Ultra-Turrax homogenizer. Samples were incubated for 2 h on ice under constant agitation. After 10 min of centrifugation at 21,000× *g* and 4 °C, the supernatants were removed. The supernatants were stored at −80 °C for further analyses, using sodium dodecyl sulfate-polyacrylamide gel electrophoresis and subsequent Western blotting on polyvinylidene fluoride (PVDF) membranes. After protein transfer, the membranes were blocked for 30 min with 5% (*w*/*v*) skimmed milk powder in TTBS (20 mM Tris, 0.1 M NaCl, 0.05 % Tween20, pH 7.5). For α-dystroglycan detection in Western blotting, a monoclonal anti-α-dystroglycan antibody (clone IIH6C4, Merck, Darmstadt, Germany) was used as primary antibody at a dilution of 1:1000 in TTBS. The incubation with this antibody was performed for 1 h at room temperature (RT) and subsequently at 4 °C overnight. As loading control, an anti-glyceraldehyde 3-phosphate dehydrogenase (GAPDH) antibody (ab8245, Abcam, Cambridge, UK) was used at a dilution of 1:10,000. As secondary antibody, anti-mouse HRP-linked antibody (554002, BD Biosciences, Franklin Lakes, NJ, USA) was used. For Western blot imaging and calculation of the apparent molecular masses, the FluorChem FC2 Imaging System (Cell Biosciences, Santa Clara, CA, USA) was used.

### 4.5. Immunofluorescence Analysis

Frozen cardiac tissue was sliced into 5 µm sections using a cryomicrotome (Leica, Wetzlar, Germany). After thawing, slides were fixed with a cooled 1:1 solution of ethanol and acetic acid. After washing with phosphate buffered saline (PBS), the slices were blocked with 8% (*w*/*v*) bovine serum albumin (BSA) in PBS for 30 min at RT. Blocked sections were incubated with anti-α-dystroglycan antibody (clone VIA4-1, Merck, Darmstadt, Germany) at a 1:100 dilution in PBS overnight at 4 °C. After washing in PBS, the slices were incubated with goat anti-mouse Alexa Fluor^®^ 488 conjugate antibody (ThermoFisher Scientific, Waltham, MA, USA) at a concentration of 1:100 in PBS for 1 h at RT. Afterward, the slices were washed and incubated with 1µg/mL 4′,6-Diamidine-2′-phenylindole dihydrochloride (DAPI)-solution (Carl Roth, Karlsruhe, Germany) in BSA/PBS for 5 min at RT. The sections were washed with distilled water and embedded using Mowiol 4-88 (Carl Roth, Karlsruhe, Germany). Image acquisition was performed using the TCS SP8 confocal microscope (Leica, Wetzlar, Germany).

### 4.6. Molecular Modelling

Structures for the wildtype and mutant fukutin were calculated using ColabFold [23] on the default settings. Structures were refined with Amber-Relax. For the wildtype and mutants, the structure with the highest pLDDT was chosen. The structures were aligned, and polar contacts were calculated for the wild type and mutant residues to other atoms in the object with PyMOL Molecular Graphics System 2.5.2 (Schrodinger, LLC, New York, NY, USA).

## Data Availability

Not applicable.

## Supplementary Materials

The following supporting information can be downloaded at: https://www.mdpi.com/article/10.3390/ijms23126685/s1.
