# Supplementary material for: Compound Heterozygous FKTN Variants in a Patient with Dilated Cardiomyopathy Led to an Aberrant α-Dystroglycan Pattern
Source: Int J Mol Sci. 2022 Jun 15;23(12):6685. doi: 10.3390/ijms23126685 (PMC9223741; doi:10.3390/ijms23126685)
Supplement: Supplementary file 1 [file ijms-23-06685-s001.zip › ijms-1747271-supplementary.pdf]

Table S1: Genotype of the index patient (III.3) analysed with panel sequencing. Only variants with a minor allele frequency < 0.0005 in GnomAD [1] are shown. As *SNTA1* is only listed with Long QT syndrome in the Online Mendelian Inheritance in Man database (https://www.ncbi.nlm.nih.gov/omim) the cosegregation of this variant within the family was not analyzed.

**gene**	**reference sequence**	**variant**
FKTN	NM_006731.2	c.895A>C, p.Ser299Arg, rs367662190
FKTN	NM_006731.2	c.1325A>G, p.Asn442Ser, rs1429464723
SNTA1	NM_003098.2	c.356A>G, p.Lys119Arg, rs n.a.

1. Karczewski, K.J.; Francioli, L.C.; Tiao, G.; Cummings, B.B.; Alfoldi, J.; Wang, Q.; Collins, R.L.; Laricchia, K.M.; Ganna, A.; Birnbaum, D.P.; et al. The mutational constraint spectrum quantified from variation in 141,456 humans. *Nature* **2020**, *581*, 434-443, doi:10.1038/s41586-020-2308-7.
